# Forma mentis networks quantify crucial differences in STEM perception between students and experts

**DOI:** 10.1371/journal.pone.0222870

**Published:** 2019-10-17

**Authors:** Massimo Stella, Sarah de Nigris, Aleksandra Aloric, Cynthia S. Q. Siew

**Affiliations:** 1 Institute for Complex Systems Simulation, University of Southampton, Southampton, United Kingdom; 2 Complex Science Consulting, Lecce, Italy; 3 Institute for Web Science and Technologies, University of Koblenz-Landau, Koblenz, Germany; 4 Scientific Computing Laboratory, Center for the Study of Complex Systems, Institute of Physics Belgrade, Belgrade, Serbia; 5 Department of Psychology, University of Warwick, Coventry, United Kingdom; 6 Department of Psychology, National University of Singapore, Singapore, Singapore; University of Sao Paulo, BRAZIL

## Abstract

In order to investigate how high school students and researchers perceive science-related (STEM) subjects, we introduce *forma mentis networks*. This framework models how people conceptually structure their stance, mindset or *forma mentis* toward a given topic. In this study, we build forma mentis networks revolving around STEM and based on psycholinguistic data, namely free associations of STEM concepts (i.e., which words are elicited first and associated by students/researchers reading “science”?) and their valence ratings concepts (i.e., is “science” perceived as positive, negative or neutral by students/researchers?). We construct separate networks for (*N*_*s*_ = 159) Italian high school students and (*N*_*r*_ = 59) interdisciplinary professionals and researchers in order to investigate how these groups differ in their conceptual knowledge and emotional perception of STEM. Our analysis of forma mentis networks at various scales indicate that, like researchers, students perceived “science” as a strongly positive entity. However, differently from researchers, students identified STEM subjects like “physics” and “mathematics” as negative and associated them with other negative STEM-related concepts. We call this surrounding of negative associations a negative *emotional aura*. Cross-validation with external datasets indicated that the negative emotional auras of physics, maths and statistics in the students’ forma mentis network related to science anxiety. Furthermore, considering the semantic associates of “mathematics” and “physics” revealed that negative auras may originate from a bleak, dry perception of the technical methodology and mnemonic tools taught in these subjects (e.g., calculus rules). Overall, our results underline the crucial importance of emphasizing nontechnical and applied aspects of STEM disciplines, beyond purely methodological teaching. The quantitative insights achieved through forma mentis networks highlight the necessity of establishing novel pedagogic and interdisciplinary links between science, its real-world complexity, and creativity in science learning in order to enhance the impact of STEM education, learning and outreach activities.

## Introduction

Increasing evidence indicates that many students develop a negative perception of STEM subjects before ending high school [1–3]. Mathematics is viewed as a difficult subject, physics is perceived as too abstract, and statistics is often considered an uninterpretable black box [3, 4]. A growing disinterest of students towards Science, Technology, Engineering and Mathematics (STEM) disciplines represents an unseen societal cost, as it translates into a lower interest in pursuing technological and scientific careers which are increasingly found to positively correlate with job growth, higher employment rates, societal innovation through functional literacy and economic development [5, 6]. Before addressing students’ (mis)perception of STEM subjects, educators and policymakers first need to understand the detailed nature of the students’ opinions and beliefs about science.

With this aim, this paper capitalizes on an innovative combination of methods from network science and cognitive science to examine the perception of STEM subjects among a population of students and another population of researchers. Specifically, we introduce the methodology of *forma mentis networks* (FMNs), which represent the associative structure of concepts as well as their valence, and show how FMNs can be harnessed to study a population’s stance toward a given topic.

Forma mentis networks are constructed from language data. Linguistic information, such as text or speech, often conveys the opinion or attitude of an individual towards a given entity [7, 8], e.g., a human reading a blog can understand which posts are in favor or against a given political view. However, stance detection, i.e., detecting the stance of an individual or population from language [9, 10], is not an easy task.

In the past few decades, stance detection has spurred research at the interface of psycholinguistics and computer science, which has led to the development of a variety of methodologies through the human coding of grammatical features of text [9, 11], e.g. the use of specific adverbs or writing styles. Such approaches are centralised, in that they require a human coder, e.g. a linguist, to parse the input and detect the features that are important for the identification of the author’s stance.

Centralised human coding cannot deal with the large volumes of linguistic data that are increasingly available, for instance from social media platforms [12]. This motivated the development of automatic techniques for detecting stance based on computer science approaches such as machine learning [10, 13, 14]. A notable example is a recent approach by Mohammad and colleagues [14], who deployed machine learning of sentiment features and word embeddings for successfully detecting the stance of individual messages from social media. The results of Mohammad and colleagues clearly show that stance detection is not the same as sentiment analysis. Sentiment analysis determines the specific affect valence of a given piece of linguistic data, i.e. how universally positive/negative/neutral are the concepts elicited by a given portion of text. Instead, stance emerges at a higher level as a non-trivial combination of different patterns of affect and sentiment. For example, the sentence “The dictator who killed my relatives has been finally executed” includes concepts of negative sentiment (e.g. dictator, executions, etc.), but nonetheless elicits a positive stance towards the execution itself. It is important to underline the additional complexity of stance in comparison with sentiment, as affective patterns in the language need to be integrated with additional contextual information before achieving an accurate classification of stance itself [14].

Although machine learning approaches are powerful in underlining the different psychological dimensions of stance in terms of context and sentiment [14], these automatic techniques have at least two limitations: (i) performance depends on the availability and quality of large-scale annotated training data, and (ii) machine learning builds “black-box” representations of data that cannot be directly accessed or interpreted. Due to these two elements, supervised learning approaches to stance detection are not yet widespread in the cognitive sciences, although they represent an interesting and powerful perspective for future work.

Beyond supervised learning, network models stand as a promising avenue to the investigation of cognitive and linguistic data, leading to the emergence of the field of cognitive network science [15]. Network models of language are often interpreted as descriptive representations of the *mental lexicon*, a repository of linguistic and semantic knowledge in human memory [7]. Decades of research in psycholinguistics has shown that the mental lexicon is not a static list of words, e.g. a dictionary, but it rather is a dynamical system optimized for cognitive computing which stores and processes individual concepts together with their associated linguistic data, e.g. semantic overlap in meaning [16], phonological similarities [17, 18], syntactic relationships between word categories [19]. Psycholinguistic evidence has shown that the associative structure of the mental lexicon influences language processes such as word learning [20–22] and processing [16, 23–25]. This strong link between mental lexicon structure and language usage promoted the use of network models for a variety of processes such as the discovery of writing styles and text authorship from word co-occurrences in texts [26, 27], improving the accuracy of clinical diagnosis of Alzheimer’s Disease risk [28], modelling and understanding the success rates of picture naming in people with aphasia [29], predicting the creativity of individuals [30–32], their curiosity [33, 34], their openness to new experience [35], their expertise in a given domain [36, 37] and their perceived anxiety toward a topic [38]. Forma mentis networks rely on the framework of cognitive network science to represent the associative and emotional structure of concepts in the mental lexicon.

One of the main ingredients of FMNs is free association data to specify the connections between concepts. Indeed, free associations represent a powerful and meaningful way of building network models of the mental lexicon [23–25, 31]. Free associations are obtained empirically from experiments where participants have to produce associates when primed with a cue word. Hence, free associations are largely free from any specific semantic definition (e.g., synonyms). Previous work [19, 20] has shown that free associations partially overlap with other semantic word-word similarities such as synonyms (i.e., two words sharing the same meaning in a given context) or generalisations (i.e., a concept being a special type of another word) but also display a small overlap with phonological similarities among words (e.g., when pronunciations differ in one phoneme).

Forma mentis networks combine free associations with affective patterns of concepts. In a FMN, nodes represent concepts or words, links indicate free associations provided by a given population and every node has a valence attribute [39–41] that represent how the population perceives a given concept or word (positive, negative, or neutral). Recent psycholinguistic evidence has shown that the emotional valence of words influences language processing and memory [41, 42], highlighting an important link between affect and the cognitive mechanisms of language processing in the mental lexicon. Therefore, representing knowledge and sentiment combined in a forma mentis network gives access to the structure of the aggregated mental lexicon and affect of a given population.

We emphasize that the addition of valence attributes and the adoption of free associations makes forma mentis networks different from conceptual maps [43–46], which represent important network models of knowledge acquisition and structuring during learning but do not incorporate information about how learners perceive individual conceptual units. Another difference is that conceptual maps are often based on concept co-occurrence in a syllabus and therefore capture temporal information [46], which is not present in a forma mentis network.

Notice that forma mentis networks rely on free associations, which capture the associative structure of semantic memory [23–25] through an empirical assessment of which concepts quickly remind of each other. Hence, these associations mirror memory patterns and are “free” from basic communication demands in sentences, such as the need to link words according to specific syntactic rules [25]. This aspect makes free associations qualitatively different from other types of word-word relationships like word co-occurrences or syntactic dependencies [27] which rather capture syntactic relationships (e.g., a verb being related to a noun). In quantitative terms, co-occurrences and syntactic dependencies can be derived automatically from written corpora, whereas free associations usually require a behavioral experiment. Furthermore, it is important to underline that free associations might overlap with syntactic dependencies but also comprise a wider variety of conceptual associations [7, 23, 25], ranging from sound similarities to meaning overlap, from visual similarity to semantic feature sharing. Forma mentis networks build on this richness of associative knowledge for representing the mindset or *forma mentis* of groups of individuals.

In this paper, we investigated the attitude of students and researchers towards science and STEM subjects through this innovative combination of tools from psycholinguistics and network science to quantify their stances toward STEM subjects. Through the comparison of the FMN of students and research professionals, we provide quantitative evidence for sharp differences in the perception of STEM among the two different groups. Specifically, the combination of conceptual associations and affect patterns allowed us to identify and paint a richer picture of the disaffection towards mathematics and physics exhibited by students and absent in STEM professionals.

## Methods

### Participants

We collected data from 159 students and 59 researchers. Students were selected from three different Italian high schools, without consideration of their grades in STEM subjects. All students were in their final year of high school, with ages ranging between 18 and 19 years (mode: 18 years). In order to build a sample representative of the national Italian student population in high schools, entire classes were selected for testing, to ensure a mixture of socio-economic backgrounds and STEM proficiency levels. Participants were roughly evenly distributed between female (53%) and male (47%) students.

Researchers were selected from large-scale international workshops because of the necessity of physically interviewing large numbers of experts at once. Our selection focused on early career scientists, which included doctoral students and post-doctoral researchers, with the aim of including as many diverse backgrounds as possible. We focused our selection towards researchers applying quantitative tools originating in the fields of mathematics, physics and computer science to study emerging phenomena in complex systems ranging from biological to socioeconomic systems. Hence, all the interviewed researchers possessed advanced training and expertise in STEM and were actively pursuing a professional career in science. The age of the interviewed researchers ranged between 24 and 39 years, with a mode of 29 years. Participants were roughly evenly distributed between male (56%) and female (44%) researchers.

### Cognitive tasks

Each participant took part in a survey composed of two tasks: (i) a free association task and (ii) a valence evaluation task. Participants were given precise instructions about the study before proceeding. Participants were then asked to provide informed consent if they agreed to take part in the study by signing a consent form that described essential points about privacy and ethics. All consent forms were gathered at the end of the study and are available upon inquiry to the first author.

In the free association task, each participant was presented with a list of 50 cue words. In order to investigate attitudes toward key STEM subjects, 10 out of the 50 cue words were present in all participants’ lists. These words were: *mathematics*, *complex*, *physics*, *chemistry*, *system*, *biology*, *life*, *art*, *school* and *university*. In Italian, these words were translated as: *matematica*, *complesso*, *fisica*, *chimica*, *sistema*, *biologia*, *vita*, *arte*, *scuola* and *università*. Although “art” is not a STEM subject, its inclusion in the list of essential words was meant to provide some comparison between the humanities and technical subjects. Additionally, adding art to the list of essential words provides a way to probe students’ perception of connections between STEM subjects and creativity, a link that has been investigated in previous studies about attitudes towards STEM [3]. The other 40 cue words were drawn at random from a subsample of STEM-focused 390 words. The pool of 390 potential cue words was obtained by considering the highest frequency non-stop words from the Wikipedia webpages about “Complex System”, “Physics”, “Mathematics”, “Biology”, “Chemistry” and “Psychology” (as accessed on: 15 January 2017).

Participants were randomly assigned to one of the 50 files containing a different random realisation with 50 of the previously described words. The order of words in each list was scrambled with the aim of reducing recency effects or other associative biases due to the order of cues.

In order to obtain denser networks of free associations, we used the continuous free association task, which has been shown to provide higher quality data that could account for more variance in lexical retrieval tasks [23]. In the continuous free association task, each participant generated three associative responses to each item in the list of 50 cue words. The association task took place in a lab setting, with each participant filling in electronic forms on a computer terminal while under supervision. Forms that contained more than 25 percent blank responses were discarded. This occurred in roughly 2% of the cases. The first three associates in each form were discarded in order to minimize potential priming effects that might follow the given instructions. The association task lasted for 10 minutes, followed by a short break.

In the second task, we collected the valence of each cue and for all associations from the free association task. Participants were asked to rate the valence of each cue and their associated responses using a Likert scale ranging from 1 (very negative) to 5 (very positive), with neutrality being represented either by a blank space or by a score of 3. Participants completed this task in about 10 minutes. Those participants who did not finish the task in 10 minutes left the remaining spaces blank. Forms that contained more than 25 percent blank responses were discarded. This occurred in roughly 3% of the cases. Data collection was conducted anonymously, such that no demographic or educational data was obtained from the participants and directly linked to the filled forms.

### Data cleaning and network building

Associative responses were converted to lowercase letters and checked automatically and manually for common spelling mistakes. The automatic spell checkers used were based on Google Translate and Wolfram’s Mathematica 11.3 (manufactured by Wolfram Research, Champaign, US). Different word forms were manually converted to match their singular forms (e.g. in English “muscles” was changed to “muscle”) and composite responses were changed to single-word forms (e.g. in Italian “da dove” was changed to “dove”).

A forma mentis network was constructed such that nodes represented lexical items and edges indicated free associations between words. Two networks of free associations were constructed from the students’ data, one network where only associations provided by at least two different participants were considered $N_{S}^{C}$ (filtered) and a second network where no filtering was performed $N_{S}$. Given the considerably smaller sample size of researchers, only a single unfiltered network of free associations was constructed $N_{R}$. We note that considering idiosyncratic associations, i.e., associations provided by a single participant, like in $N_{S}$, is common practice when working with free association data obtained from small samples [43, 44, 47] and they are still considered to be insightful of cognitive patterns [48]. Each node in the network was also assigned a valence score and an attribute (“positive”, “neutral”, or “negative”).

In the remainder of the paper, *forma mentis network* refers to the network representation that simultaneously represents conceptual knowledge derived from free associations and the emotional perceptions of those concepts among a given population (i.e., students or researchers).

### Statistical analysis of word valence

In order to categorize positive, neutral and negative concepts we used a non-parametric statistical test (Kruskall-Wallis test). The statistical test was used to assess whether the scores attributed to word *i*, namely *w*_*i*_, had a lower, compatible, or higher median valence as compared to the remaining distribution of valence scores, in formulas ⋃_*j*≠*i*_ *w*_*j*_. Non-parametric testing was used because the original distribution of valence scores ⋃_*j*_ *w*_*j*_ were skewed with a heavy left tail (Pearson’s skewness coefficient *s*_*s*_ = 3(*mean*_*s*_ − *median*_*s*_)/*σ* = 1.39 for students’ data and *s*_*r*_ = 1.45 for researchers). Concepts which had a median valence score lower than the rest, according to a Kruskall-Wallis test with significance level *α* = 0.1, were labelled as *negative*. Concepts which had a median valence score higher than the rest, according to a Kruskall-Wallis test with significance level *α* = 0.1, were labelled as *positive*. Remaining concepts were labelled as neutral.

### Defining valence beyond lexical items: Valence “auras”

Valence is a commonly used feature in psycholinguistic models that assesses the sentiment of a given text [8, 39]. At the word level, the valence of a word represents the positive, neutral, or negative connotation is elicited by the word in a given population. Hence, valence is a feature of individual words, and does not further consider the way in which words are associated with each other.

Combining free associations with their valence in forma mentis networks naturally provides a way of extending the concept of word valence to a given cluster of associated words. We introduce the concept of *valence aura*, which identifies the valence of the immediate neighbors of a word on a network of free associations. A concept has a negative valence aura if the given concept is associated with more negative concepts than it is associated with positive concepts. On the other hand, a concept has a positive valence aura if the given concept is associated with more positive concepts than it is associated with negative concepts. The polarity of an aura is determined by the most frequent valence of words in the neighborhood (following a majority rule). It is important to note that positively valenced words could have either a negative or positive valence aura, and negatively valenced words could have either a negative or positive valence aura. Hence, valence auras provide us with a way of juxtaposing or contrasting sentiment polarities with consideration of the structural organisation of knowledge beyond how individual concepts are valenced in isolation.

We use the methodology of valence auras to investigate potential differences in the way that students and researchers structure their conceptual knowledge and the role of the perceived valence of those concepts. Firstly, within a given population, it would be interesting to assess the tendency of positive concepts to be associated with other positive concepts (i.e., to have a positive valence aura), and equivalently for negative concepts (i.e. negative concepts with other negative ones). This would bolster the idea that sentiment polarities of individual words have the potential of influencing the structural organisation of semantic memory. Secondly, and more importantly, there might be different tendencies to surround positive concepts with positive or negative auras between students and researchers. Differences in the mixing of (individual) word valence and word auras between the forma mentis networks of different populations could potentially highlight important differences in the organisation and perception of knowledge between such populations.

### Validation of the definition of auras through additional psycholinguistic data

In order to validate our operationalization of valence auras and the valence ratings collected in the present study, we used external datasets of word valence for comparison. For English words in the researchers’ forma mentis network, we used the affective ratings by Warriner and colleagues [40], whereas for Italian words in the students’ forma mentis network we used the valence norms recently gathered by Fairfield and colleagues [49]. Kendall tau correlation tests indicate that in both cases there is a statistically significant positive correlation (at *α* = 0.1) between the mean valence scores collected in the current study and the ones obtained from previous investigations (for students: *τ* = 0.51, *p* < 10^−5^; for researchers: *τ* = 0.38, *p* < 10^−5^). For students, the dataset by Fairfield et al. contained valence scores for only 491 of the 4483 words in the Italian unfiltered forma mentis network. For researchers, the overlap between our dataset and Warriner and colleagues’ was higher, covering 1173 of the 1616 words in the English unfiltered forma mentis network.

In the following, we will not use directly the mean valence scores gathered from students or researchers but rather valence attributes (i.e., positive, neutral, or negative) to define the valence auras of words. In the results section, we will show that our retrieved valence attributes are compatible with the valence scores obtained by other studies.

## Results

### Network structure and valence identify auras, which in turn identify words of extreme valence and arousal

The operationalization of valence auras as reported in the Methods section combines network structure with the valence of individual words. Does this combination of topological and valence information provide further insights into students and researchers’ perception of STEM subjects? In order to answer this question, we compared the mean valence and arousal scores from external psycholinguistic datasets (cf. Methods) of negative words with either positive or negative auras, and positive words with either positive or negative auras. Recall that auras were defined by using the valence ratings and network structures obtained from the cognitive tasks described above, whereas mean valence scores come from external sources (i.e., the Fairfield [49] and Warriner [40] databases).

**Fig 1:**
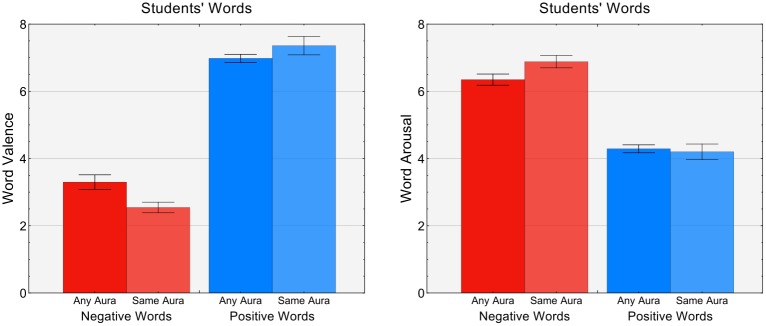
Valence auras identify more extreme negative words in the students’ population. Mean valence (left plot) from external psycholinguistic datasets of negative/positive words either surrounded by any valence auras (full colours) or by the same aura (lighter colours). In the students’ forma mentis network, negative words surrounded by negative auras are perceived as more strongly negative on average as compared to negative words surrounded by any aura. Negative words surrounded by a negative aura had higher arousal than negative words with any aura (right plot). No difference was found for positive words.

Fig 1 reports the mean valence and arousal of words from the students’ forma mentis network. In the students’ FMN, negative words surrounded by a negative aura have a lower mean valence (based on an external dataset, cf. Methods) as compared to negative words surrounded by any aura. This difference was statistically significant at the 0.1 significance level *α* (Kruskal-Wallis, *N* = 116, *s* = 2.8994, *p* = 0.088). The difference in mean valence between positive words surrounded by any aura and positive words surrounded by a positive aura was not statistically significant at the *α* = 0.1 level (Kruskal-Wallis, *N* = 238, *s* = 2.2891, *p* = 0.1314). The forma mentis network of students also highlighted an interesting difference in the mean arousal of negative words surrounded by a negative aura as compared to negative words surrounded by any aura (cf. Fig 1, left plot). Negative words surrounded by a negative aura elicited a stronger arousal as compared to negative words surrounded by any aura, and the difference was statistically significant at the *α* = 0.1 level (Kruskal-Wallis, *N* = 116, *s* = 2.7338, *p* = 0.0984). A threshold of 0.1 was chosen in order to cope with the limited overlap of our data with the external databases.

The above differences suggest that negative auras correspond to a boosted arousal when surrounding words of negative valence. Positive words did not elicit any analogous difference. No statistically significant difference was found among words in the researchers’ forma mentis network.

These results indicate that in the organisation of STEM-related concepts as represented by the forma mentis network, negative concepts surrounded by a negative aura are in general perceived as more negative and elicit higher arousal than concepts surrounded by any aura. Since the arousal scores might be a by-product of valence in rating experiments [41], negative words surrounded by a negative aura represent negative concepts that can activate or be activated by other negative concepts and thus lead to an increase in arousal and emotional intensity. The above analysis presents quantitative evidence showing that our operationalization of valence auras of an individual word’s direct neighbors in an associative network can highlight additional affective patterns that valence scores of individual words cannot.

The unfiltered forma mentis network of researchers is smaller, less connected and has fewer negative words than the students’ forma mentis network. Hence, no significant differences were found at the *α* = 0.1 level of significance (Kruskal Wallis, for positive words: *s* = 1.0674, *p* = 0.3033; for negative words: *s* = 0.0574, *p* = 0.8119). We hypothesized that this is due to the forma mentis network of researchers lacking the resolution or power to detect distinct affective patterns.

### Forma mentis networks show assortative mixing of word valence

As described above, in a forma mentis network, each word has a valence attribute (e.g. “positive” or “negative”). Links represent associations between two concepts but also between their respective valence attributes (e.g., there can be a link connecting a “positive” concept to a “negative” concept). Investigating the assortative or disassortative mixing of valence attributes across network links can shed light on potential trends in students’ and researchers’ structural and emotional organisation of knowledge.

In the unfiltered network of student associations $N_{S}$, a Kendall Tau test between the valence attributes of links’ endpoints reveals a statistically significant positive correlation *τ* = 0.163, *p* < 10^−5^. This value might indicate that students tend to associate positive (negative) concepts to other positive (negative) concepts. However, comparison with a reference null model is necessary in order to assess the relative strength of the above correlation and test whether it might be a direct consequence of either the distribution of node degree or the counts of positive, neutral, or negative attributes. We used as null reference a configuration model fixing both the empirical degree distribution and the valence attributes of words in the original network but randomising links. An average Kendall Tau of *τ*_*r*_ = −0.0001 (*p* > 0.310) was obtained over 50 independent realisations of the null model. As the empirical correlation *τ* = 0.163 was several orders of magnitude larger than random expectation, this indicated that there was a strong tendency for students to associate positive (negative) concepts with other positive (negative) concepts independently of the distributions of either degree or valence attributes. We found a similar pattern in the way that researchers organised and perceived their STEM knowledge (empirical Kendal Tau *τ* = 0.116, *p* < 10^−5^, reference null model *τ*_*r*_ = 0.027, *p* > 0.112).

### Forma mentis networks indicate clustering of word valence and valence auras

In this section we investigated whether there was a tendency for words to be surrounded by other words with the same valence. To do this, we attributed arbitrary scores to valence attributes, i.e. −*m* to negative words, 0 to neutral words and +*m* to positive words. We used *m* = 1 for convenience, although our correlation analysis does not depend on the specific value of *m*.

In the students’ forma mentis network $N_{S}$, a Kendall Tau test between the valence attributes of a word and the average valence attributes of its neighbors revealed a statistically significant positive correlation *τ* = 0.385, *p* < 10^−5^. With 50 independent realisations of a configuration null model with fixed word attributes and degrees but random associations, we found an average correlation of *τ*_*r*_ = 0.053 (*p* = 0.060). A similar result was found for the researchers’ forma mentis network $N_{R}$, where the empirical correlation value (*τ* = 0.323,*p* < 10^−5^) was considerably higher than random expectation (*τ*_*r*_ = 0.060,*p* = 0.056).

Given that the empirical correlations were several orders of magnitude larger than the reference values, our results showed a tendency for both students and researchers to associate words of a given valence with auras of the same valence, i.e., negative concepts tend to have a negative aura whereas positive concepts tend to have a positive aura. This indicates that the forma mentis networks of researchers and students are on average highly clustered in neighborhoods of words with similar valence attributes. Deviations from this general trend, such as negative words in the positive aura of a positive word, can be informative of the way in which a given population perceives STEM subjects.

### Forma Mentis networks highlight differing stances towards STEM subjects

**Fig 2:**
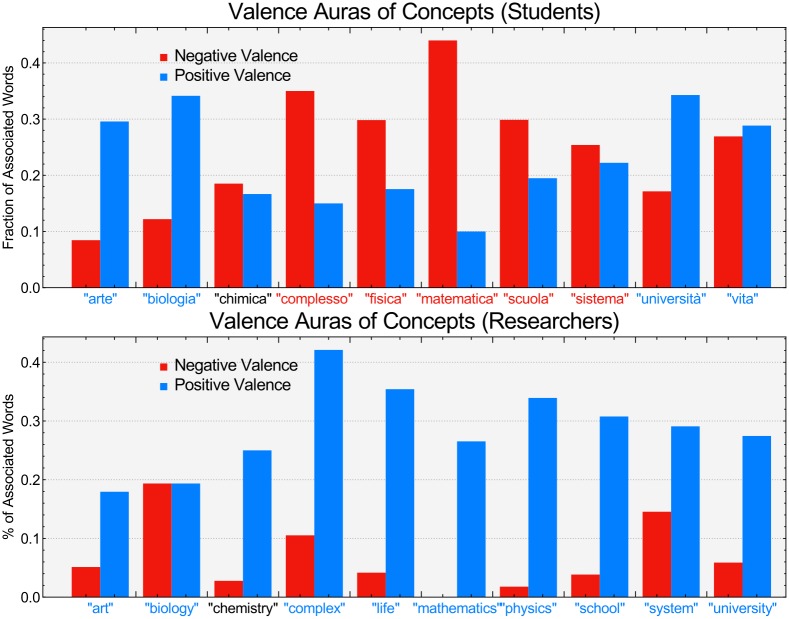
Valence auras identify a negative stance of students towards specific STEM subjects. **Top panel**: Fraction of neighbors of concepts having a positive or a negative average valence. Concepts with positive valence are reported in blue. Concepts with negative valence are reported in red. Students perceive quantitative scientific subjects such as Mathematics and Physics negatively. Students also attributed a negative aura to these disciplines, i.e., they associated Physics mainly with other negative, rather than positive, concepts. The auras of negativity were not aimed towards all STEM subjects, since biology was perceived as positive and surrounded by an aura of positive valence. **Bottom panel**: Valence auras for researchers. Notice that all essential concepts were perceived as positive and surrounded by auras of positive valence.

In this section we focus our attention on the semantic content of associations. Fig 2 reports the attribute and aura of the 10 words that were always provided to participants as cues (see Methods). Positive (negative) words are highlighted in cerulean (red) for both students (top panel) and researchers (lower panel). Researchers associated STEM-related words with mainly positive concepts, whereas students associated STEM-related words with both positive and negative concepts.

The analysis of individual words reveals that researchers perceived almost all the 10 STEM-related words as positive concepts. On the other hand, students perceived words such as “complex”, “physics”, “mathematics”, “school” and “system” as negative concepts. Furthermore, the network structure of forma mentis networks highlighted additional critical differences in the way that students and researchers attributed positive or negative auras of valence to such negatively perceived STEM words. Specifically, students associated concepts such as “physics” and “mathematics” to other negative concepts, surrounding STEM words of quantitative disciplines with a negative valence aura. The ratio of negative to positive concepts is particularly high in the case of “mathematics”, where almost 43% of associations were to other negative concepts. These patterns were absent in the forma mentis network of researchers. This comparison suggests that the presence of negative auras attributed to some STEM-related concepts is not merely a consequence of the network construction but reflects the negative stance that students have towards quantitative disciplines such as physics and mathematics.

**Fig 3:**
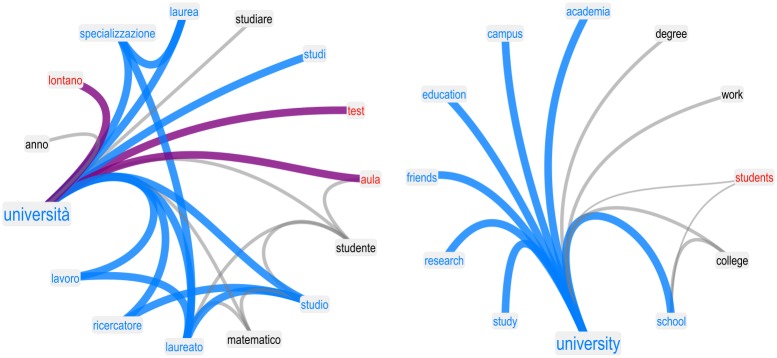
Neighborhoods in forma mentis networks determine the valence aura of concepts. Examples of the forma mentis networks in the neighborhood of “university” for students (left) and researchers (right). In a forma mentis network, nodes have valence attributes, i.e. “positive” (cerulean), “neutral” (grey) and “negative” (red). Links are weighted based on the number of participants providing a given association between concepts. Links between positive (negative, neutral, opposing) concepts are cerulean (red, grey, purple). The above examples include only associations provided by at least two participants. The neighbors surrounding a given word, together with their valence attributes, constitute the valence aura of that word. Both students and researchers perceive “university” as a positive concept and surround it with a positive aura.

However, it is worth noticing how the data also indicates that students did not perceive all STEM subjects as negative. In fact, concepts such as “biology” and “university” were perceived as positive and were surrounded by a positive valence aura in the students’ forma mentis network. This contrast suggests that the aversion of STEM-related concepts might be related to the quantitative disciplines that underlie the scientific method used in these fields. Fig 3 shows the neighborhoods of “university” in the filtered forma mentis network of students (left) and researchers (right). Notice that “university” is perceived as positive and surrounded by other positive concepts such as “degree”, “study”, “work”, and “specialisation”. Even the word “researcher” is positively perceived by students, indicating a positive stance towards the general concepts of research and education. Importantly, students strongly associated the concepts of “studying” and “work”, as indicated by the presence of this connection in the statistically filtered FMN. This result suggests that students might be aware of the positive impact of education has with respect to future success in the job market [5, 6].

**Fig 4:**
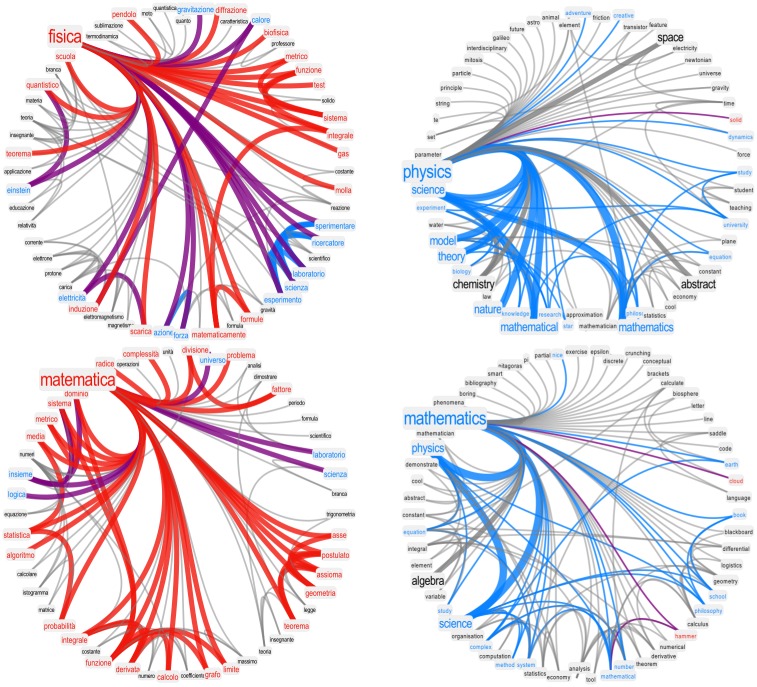
Mathematics and physics are perceived differently by students and researchers. The neighborhoods of “physics” (top) and “mathematics” (bottom) for students (left) and researchers (right). Red links indicate associations between concepts of negative valence. Stronger, more frequent associations are thicker. Students not only perceive “mathematics” and “physics” as negative concepts but also surround them with strongly negative auras of valence. This phenomenon is absent in the forma mentis network of researchers, indicating a critical negative attitude of students towards STEM quantitative subjects. Notice that for both physics and maths in students the negative aura comes mainly from clusters of specific concepts relating to specific tools (e.g. “derivative”, “test”, “integral”).

Fig 4 shows the neighborhoods of “physics” (top) and “mathematics” (bottom) in the filtered forma mentis network of students (left) and the FMN of researchers (right). Notice how concepts such as “physics” or “mathematics” gave rise to mostly negative associations in the students’ population. The hierarchical edge bundling visualisation implemented in Mathematica 11.3 highlights that negative associations tend to cluster together, in agreement with the above clustering analysis. An inspection of the semantic content of the neighborhood for “mathematics” reveals the presence of clusters of negative concepts associated with the topic of calculus and geometry. Interestingly, most of these negative concepts were concrete tools and methodologies used in mathematics (e.g. “algorithm”, “derivative”, “graph”, “theorem”), rather than abstract, more general terms such as “complexity”. A similar result holds for “physics” (e.g., “function”, “test”, “integral”). A closer look at the semantic information embedded in the negative aura surrounding “physics” and “mathematics” provides preliminary evidence that the negative perception students have of these subjects may come predominantly from a negative perception of the quantitative tools usually taught in schools. In other words, the negative aura surrounding “mathematics” or “physics” does not come from a general negativity towards the whole educational system but rather from specific, concrete elements of teaching curricula. Improving the appreciation of students towards these concrete tools (e.g. “algorithm”, “graph”, “function”) might have a beneficial effect on the perception that students have of “mathematics” or “physics”, given the average trend reported above indicating that positive concepts tend to be surrounded by positive auras. However, it is important to note that our study by itself cannot either prove or disprove a causal link about concepts being perceived as positive because of their positive auras and additional research is required.

**Fig 5:**
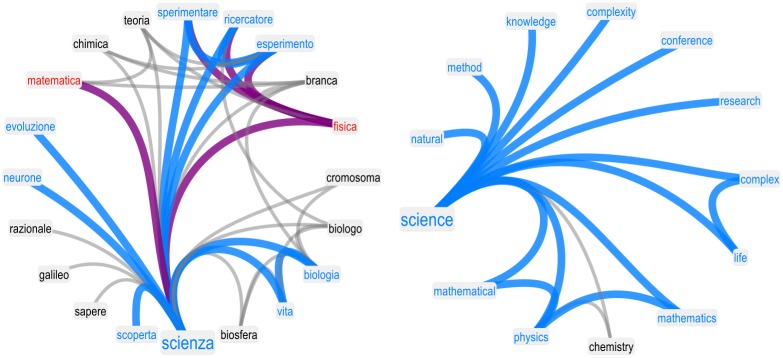
Maths and physics are the main negative outliers in the otherwise positive aura of “science” as perceived by students. “Science” was never provided as a cue either to students or to researchers. It was one of the associations provided by participants. Neighborhoods of “science” in the forma mentis networks of students (left) and researchers (right): although students perceived science and other STEM subjects as positive concepts, surrounding science itself with an aura of positive valence, students also perceived mathematics and physics as negative concepts. This plot included only statistically significant free associations.

However, it is important to underline that although “mathematics” and “physics” displayed negative auras, in both the unfiltered and filtered forma mentis networks students were able to associate these subjects to “science”, which is perceived as a positive concept (see also Fig 5). This link may indicate that students are aware of the importance of quantitative disciplines for the advancement of science.

Forma mentis networks further highlight the critical negative perception of students towards “mathematics” and “physics”. As reported in Fig 5, those are the only negative concepts in the otherwise positive valence aura surrounding “science”. This contrast indicates that, although on a technical level students were aware about the links between quantitative disciplines and science, they were unable to transfer their positive perception of science to the building blocks of the scientific method.

## Discussion

Forma mentis networks represent an innovative combination of free associations, i.e. words that are elicited in response to cue words [48], with additional affective information of each word’s valence [39, 40], i.e. how positively or negatively a given concept is perceived.

From a methodological perspective, this combination of two sources of linguistic information fills a gap in the literature of language networks modelling the mental lexicon [15], where conceptual units are considered only in terms of their semantic features [16, 23, 25, 30, 31], and not their valence. Given that recent evidence indicates that emotions deeply influence language processing and memory even at nonconscious levels [41, 42], forma mentis networks represent a natural extension of semantic representations of the mental lexicon that includes affective attributes of individual concepts.

The combination of sentiment and semantic structure leads to the definition of valence auras, in which concepts are not isolated affective entities but rather interacting emotional elements of an associative network. The empirical evidence reported in this work shows a tendency for concepts of a given valence to cluster with words of the same valence. This assortative mixing of links is known as homophily in social network analysis [50] and represents a tendency for units to link mainly to other units sharing similar features. To the best of our knowledge, our work represents the first evidence of *emotional homophily* in the human mental lexicon. This emotional homophily leads to concepts of a given valence being surrounded by other concepts with the same valence. By cross-validating our data with independent datasets of affective norms [40, 49], we showed that negative words surrounded by a negative aura elicited a higher arousal compared to negative words surrounded by any (neutral, negative, or positive) aura. This difference could be interpreted in terms of individual concepts exerting an influence over their associated neighbors, with negative words increasing levels of emotional intensity elicited during cognitive processing such that negative words surrounded by other negative words have higher arousal ratings than expected. This interaction between emotional processing and the structure of semantic memory itself should be further explored in future psycholinguistic research.

In the present paper, emotional homophily in forma mentis networks provides us with new ways of detecting the stance of a given population. Our application of the forma mentis networks has led to three main insights into the way that students and researchers perceive STEM topics.

First, we used the network structure of FMNs to identify and define the “aura” of a concept, i.e., its first neighbors in the association network created by the participants. An analysis of valence auras, in addition to the words’ individual valence attributes, uncovers a clear pattern in the students’ FMN in which negative words surrounded by a negative aura were correlated with higher arousal ratings. Moreover, words of the same valence tended to cluster together, indicating a conceptual organization that may have been shaped by the valences of words. Overall, it appeared that the students’ stance towards STEM subjects is mixed, combining both positive and negative stances, whereas the researchers’ stance toward STEM was predominantly positive.

Second, at the semantic level, the comparison between students’ and researchers’ networks led both to unexpected similarities as well as interesting contrasts. On the one hand, Italian high school students are almost as skilled as experts in relating key concepts of STEM subjects to “science”, such that “science” itself was a key concept in their forma mentis network. This finding provides evidence that at a global level Italian students possess a good technical awareness of STEM subjects in comparison with STEM professionals. Analogously, a comparable level of student competence has also been reported in other educational systems, such as the Finnish one, by independent studies from Koponen and Nousiainen using concept maps [46].

Furthermore, students perceived concepts such as “mathematics” and “physics” not only as negative, but surrounded by negative auras as well, whereas words such as “science” were positively perceived by both students and researchers. This dichotomy between the positive aura of science and the negative auras of mathematics and physics is absent in the group of STEM professionals, and it suggests that students might not be sufficiently aware of the connections between science, its methods and its applications. Another interpretation of the negative auras surrounding mathematics, physics and other concepts like statistics (cf. S1 Appendix) might relate to emotional homophily and anxiety. As discussed above, negative concepts surrounded by a negative aura tended to also have higher arousal and lower valence ratings (based on external datasets). In the circumplex model of emotions [39], higher arousal and negative valence correspond to emotions of stress and anxiety. Hence, the negative emotional auras of physics, maths and statistics in the forma mentis of students suggest that high school students may experience stress and anxiety toward such disciplines. This finding is supported by an increasingly developing literature about mathematics anxiety [4], physics anxiety [51] and even statistics anxiety [38] affecting students’ learning at the high school level and continuing even through university. Notice that emotional auras and anxiety represent a crucial problem in Education, since recent results show that stress and anxiety inhibit the acquisition and retention of STEM-related concepts [52]. When interpreted against the relevant literature, our results concretely point out the urgency for identifying and acting upon negative auras/perceptions in student populations in order to enhance STEM learning. Forma mentis networks represent an innovative way of quantifying science anxiety in student populations, potentially working in synergy with other network studies quantifying anxiety levels with complex networks [38] and psychological methodologies [4].

In spite of the bleak perception of mathematics and physics, the students showed a positive perception of both “university” and “researcher”, which indicates an awareness of the importance of education for their professional future.

Third, valence auras allowed us to hypothesize viable reasons for the disaffection towards mathematics and physics beyond anxiety: Words with a negative valence in those subjects’ aura are mostly mathematical tools and techniques, such as “integral” or “function”. This result highlights how the negative stance on physics and mathematics might not originate from a wider distrust of the subjects themselves but perhaps from the difficulty in seeing the value of these techniques, particularly when devoid of interdisciplinary connections. This result opens possible avenues for intervention in education by, for instance, helping students to embed mathematics and physics within a richer network of conceptual associations.

What might be missing from the educational curriculum of the students participating in this study is an emphasis on the connections between quantitative disciplines and real-world settings. Beyond sterile arguments that a discipline should be appreciated because of its inner beauty, our results suggest that even at the high school level, educators should provide as many opportunities as possible for students to discover the beauty of STEM subjects and learn about the implications of mathematical modelling of real-world systems, as previously highlighted in the relevant educational literature [45, 47, 53, 54].

A positive stance towards mathematics and physics among early career complexity researchers could be due to the fact that many real-world models of complexity science are grounded in quantitative disciplines such as mathematics and physics [53, 55]. However, a large proportion of complex systems scientists do not identify as mathematicians or physicists, and come from a diverse range of disciplines, including biology, economics, chemistry, archaeology, art, psychology, and the social sciences. The application of quantitative tools to aid the understanding of complex systems might have led to a positive perception of these concepts among professionals, as reflected in their forma mentis network.

Complexity science seems to be a natural candidate for improving the perception of STEM subjects among students. Indeed, previous attempts at building network science courses at the high school level are generally met with interest by high school students [47, 54, 56]. In addition, the Complex Forma Mentis project (www.complexmentis.com Last Accessed: 19 February 2019) provided seminars about complexity science at the high school level that were met with strong interest. Although these initiatives are still early in their implementation, the framework of complexity science may prove to be useful in helping students learn about how the technical aspects of STEM disciplines can be used to address important societal problems, and as a result improve their perception of technical, sometimes obscure, concepts related to mathematical theory and physics.

Another reason behind the dissonant stance of students towards physics and mathematics might be related to a lack of creativity. Recently, Valenti and colleagues [3] measured the implicit attitudes towards science in a population of students and found that the increase of scientific rigour is accompanied by a decrease in associating science with creativity. In the FMN of researchers, “art” is connected to “creative” and “science”, whereas these concepts were disconnected in the forma mentis network of students. Researchers also associated “physics” with “creative”, an association that is missing in students’ FMN. These missing links further underline the importance for students to build a more complete and broader perception of STEM subjects, focusing on the creative process behind science and its real-world, complex implications. Creating such links in high school students, even through simple actions like complexity-focused outreach events, represents a practical task outlined by our results of utmost importance for improving STEM perception.

Nevertheless, the current approach of FMNs has some limitations that we discuss below. The most prominent one is that forma mentis networks operate at the population level, as is common in psycholinguistic approaches relying on free association networks [23, 48] or in educational studies using concept maps [43–45]. Hence, the above patterns have to be interpreted in terms of average trends, as individual students might differ from the aggregated pattern. However, recent approaches have constructed association networks at the level of individuals [31, 36] and even reported how individualized free association networks were predictive of creativity levels [31] or knowledge mastery [36]. With larger sample sizes and a more substantive free association task (leading to denser networks), building forma mentis networks for the individuals represents an exciting research direction for the future.

Another limitation is the experimental effort in engaging participants within a cognitive task, compared to the relative ease of mining online data from social media in order to infer stance. A possible solution could be the use of social media mining to extract semantic associations for forma mentis networks, analogous to the semantic networks of concept co-occurrences in Twitter by [12]. Although this might decrease the difficulty of building a network representation of the mental lexicon of a given population, co-occurrences of words in text are different from free associations and provide different cognitive information with regards to language acquisition and use. For instance, in [20], free associations proved to be more predictive of early word learning compared to word co-occurrences in child directed speech. Hence, despite the difficulty of collection free associations, we argue that free associations provide important insights into the structure of the mental lexicon and that such data are worth the time and cost of data collection.

### Potential impact of forma mentis networks in education and beyond

Forma mentis networks represent a powerful new framework that can help tackle important research question within Education research and beyond.

We envision that the most useful educational utilisation of FMNs lies in learning assessment and data-driven educational policy making. The cognitive representation of students’ mindsets provided by FMNs represents a powerful way of testing the impact and effectiveness of different teaching methods. Comparisons between the FMNs of a class of students before and after attending a course could provide global and microscopic quantitative information about how students changed their perception and deep understanding of course topics. Furthermore, individual FMNs could be built and correlated with course grades in order to assess the most beneficial changes in mindsets correlating with best exam performances. Promising language network applications of this type have been recently suggested [36, 37, 46] and they confirm the power of network-based representations of knowledge for performance assessment beyond standard tests or quizzes. Once corroborated against individual-level learning performances, FMNs would provide a data-informed approach for facilitating and accelerating conceptual learning based on learners’ mindsets.

Forma mentis networks constitute a novel representation of conceptual knowledge and as such can be of great relevance for the understanding of cognition and information processing beyond educational setting. Recently, networks of conceptual knowledge have proved valuable models for understanding knowledge building and exploration in relation to personality traits such as curiosity, openness to experience and creativity [33, 57]. Modelling knowledge acquisition through the statistical mechanics of network walks, de Arruda and colleagues [57] showed that on artificial networks of conceptual associations, knowledge building is consistently stronger in central network regions, i.e. for tightly connected concepts connected by a few steps to all other words. Testing the same dynamics over a “real” mindset represented by a FMN would further characterize the relevance and meaning of key concepts in a given forma mentis. For instance, how relevant “robotics” and “health” can be in healthcare knowledge building across different groups of medical professionals?

Notice that knowledge creation has been recently shown to be driven not only by conceptual centrality/relevance but also by individual personality aspects, such as curiosity (i.e., the proclivity to search for information). It would be interesting to investigate whether the structure of individual FMNs correlate with longitudinal data about curiosity levels. This would allow us to identify distinctive features in mindset organisation around specific topics in high and low curiosity people. Building on the powerful approach by Lydon-Staley and colleagues [34], who recently found that higher curiosity corresponds to tighter networks of associations, FMNs would offer the possibility to assess the impact that positive/negative/neutral concepts and emotional auras play in knowledge building across various levels of curiosity. Another personality trait investigated through associative network approaches was Openness to Experience, the enjoyment of novel ideas and experiences. Christensen and colleagues [35] showed that groups of individuals with a higher Openness to Experience gave rise to a more interconnected network of conceptual associations, thus opening new challenges for characterizing such personality trait in terms of network data. Formulating a predictor of Openness to Experience relying on the conceptual knowledge and sentiment patterns encapsulated in a FMN would complement the descriptive power of forma mentis networks in outlining the stance, and acceptance, a given group has toward a given topic. Another interesting interplay to investigate with FMNs would be the one between knowledge structure and creativity levels. Several recent network approaches managed to correlate the structure of semantic memory with creativity levels [30, 31]. At a population level, Stella and Kenett [32] showed that the multiplex combination of free associations with other semantic, categorical and phonological word-word relationships identifies a central region in the network of conceptual knowledge. The authors found that high/low creativity level people accessed this central region in several different ways. The authors exploited these differences for implementing a machine learning predictor of creativity levels. Replacing the layer of free associations with a forma mentis network and following the protocol by [32] would enable novel ways of testing how creativity levels impact knowledge exploration for different, specific mindsets. Also, building upon our above findings about negative emotional auras correlating with anxiety eliciting and anxiety being a distinctive trait of creative people [8], this application could shed more light on the interplay between the emotional homophily detected in this work, negative/positive sentiment and knowledge exploration across high and low creativity levels.

## Conclusions

This article introduced the new methodology of forma mentis networks and demonstrated its potential to identify contrasting stances in different populations. A forma mentis network consists of words as nodes, each with a valence attribute, and free associations as links. Rather than being based on automatic natural language processing, these networks directly access the mental lexicon of human participants, addressing the orthogonal influences of semantic knowledge and emotional affect that drive the processing of information [15, 17, 18] and its consequences [33, 35, 41, 42].

We found substantial differences in the stances of young researchers in complexity science and high school students towards STEM concepts such as physics and mathematics. Students tended to surround these concepts with a negative emotional aura, which could related to a perceived anxiety toward these subjects (cf. [4, 38, 39]). This negative emotional aura was absent in the forma mentis of researchers. Furthermore, the words with a negative valence in the students’ neighborhoods were mostly that of mathematical tools, such as “integral” or “function”. This result highlighted how the students’ negative stance toward physics and mathematics might predominantly originate from an arid view of the tools and methods used in mathematics and physics, which students (but not researchers) perceived as deprived of more interdisciplinary and creative connections.

This quantitative evidence opens new avenues for intervention in education: Encouraging students to incorporate mathematics and physics into a richer association network and drawing new connections to other concepts in the scientific realm represent promising pathways to change their stance. In that lies the potential of the forma mentis network approach–by providing a map of the students’ mental lexicon, it is able to show which and where new meaningful links, i.e. associations, could be constructed to maximize the effectiveness of future intervention policies and outreach programmes.

## Supporting information

S1 Appendix Supporting text and figures. (PDF)
