# Supplementary material for: Forma mentis networks quantify crucial differences in STEM perception between students and experts
Source: PLoS One. 2019 Oct 17;14(10):e0222870. doi: 10.1371/journal.pone.0222870 (PMC6797169; doi:10.1371/journal.pone.0222870)
Supplement: S1 Appendix — (PDF) [file pone.0222870.s001.pdf]

Supporting Information for Forma mentis networks quantify crucial differences in STEM perception between students and experts

Massimo Stella[1,2*], Sarah de Nigris[3], Aleksandra Aloric[4], Cynthia S. Q. Siew[5,6]

1 Institute for Complex Systems Simulation, University of Southampton, Southampton, UK
2 Complex Science Consulting, Lecce, Italy
3 Institute for Web Science and Technologies, University of Koblenz-Landau, Koblenz, Germany
4 Scientific Computing Laboratory, Center for the Study of Complex Systems, Institute of Physics Belgrade, Belgrade, Serbia
5 Department of Psychology, University of Warwick, Coventry, UK
6 Department of Psychology, National University of Singapore, Singapore

* Corresponding author: massimo.stella@inbox.com

This Supporting Information outlines the role played by idiosyncratic associations in forma mentis networks, i.e. associations there were provided by less than two different participants in the free association task. When using free associations for modelling the structure of the mental lexicon, idiosyncratic associations are usually filtered out and the focus is given to more frequent associations between concepts [?]. However, also idiosyncratic associations can provide additional information about how individuals perceive the external world [?], given that associations' idiosyncracy might be just an outcome of the limited number of interviewed participants in a given free association task. Hence, also idiosyncratic associations can be relevant for the above investigation of stance from FMNs.

S1 Fig. reports forma mentis networks of students and researchers in the neighborhood of "university" including also idiosyncratic associations. In the students' forma mentis network, important concepts like "courses", "curricula" or "academia" are linked to "university" only when idiosyncratic associations are considered.

S2 Fig. reports the neighborhood of "science" in the students' FMN including also idiosyncratic associations. Even when the noise introduced by idiosyncratic associations is considered, science is mostly surrounded by a positive emotional aura, where negative concepts are mainly those related or clustering around physics and maths. Hence, idiosyncratic associations do not alter the emotional gap discussed in the main text about STEM stance.

S3 Fig. reports the neighborhood of "statistics" in the students' and researchers' FMNs including also idiosyncratic associations. Beyond mathematics and physics, also other STEM concepts like statistics are surrounded by a negative aura in the students' FMN, a negative perception that is absent in the researchers' forma mentis network.

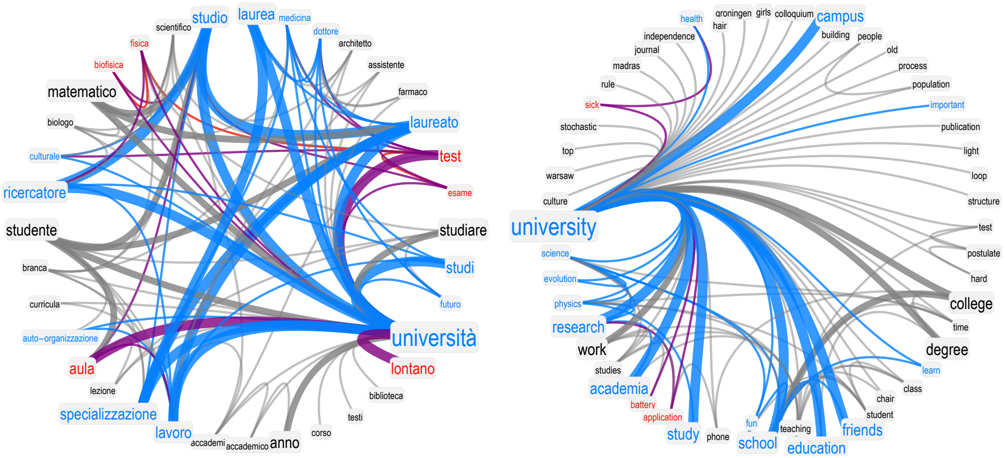

Fig 1. Idiosyncratic interactions provide additional network structure. Examples of the unfiltered forma mentis networks in the neighborhood of "university" for students (left) and researchers (right), including also idiosyncratic associations made only by one participant. Associations provided by two or more participants are marked thicker, with the same colour scheme of Figure 3 in the main text. Notice that some associations might be idiosyncratic only because of the smaller pool of participants and they might still provide meaningful additional semantic content.

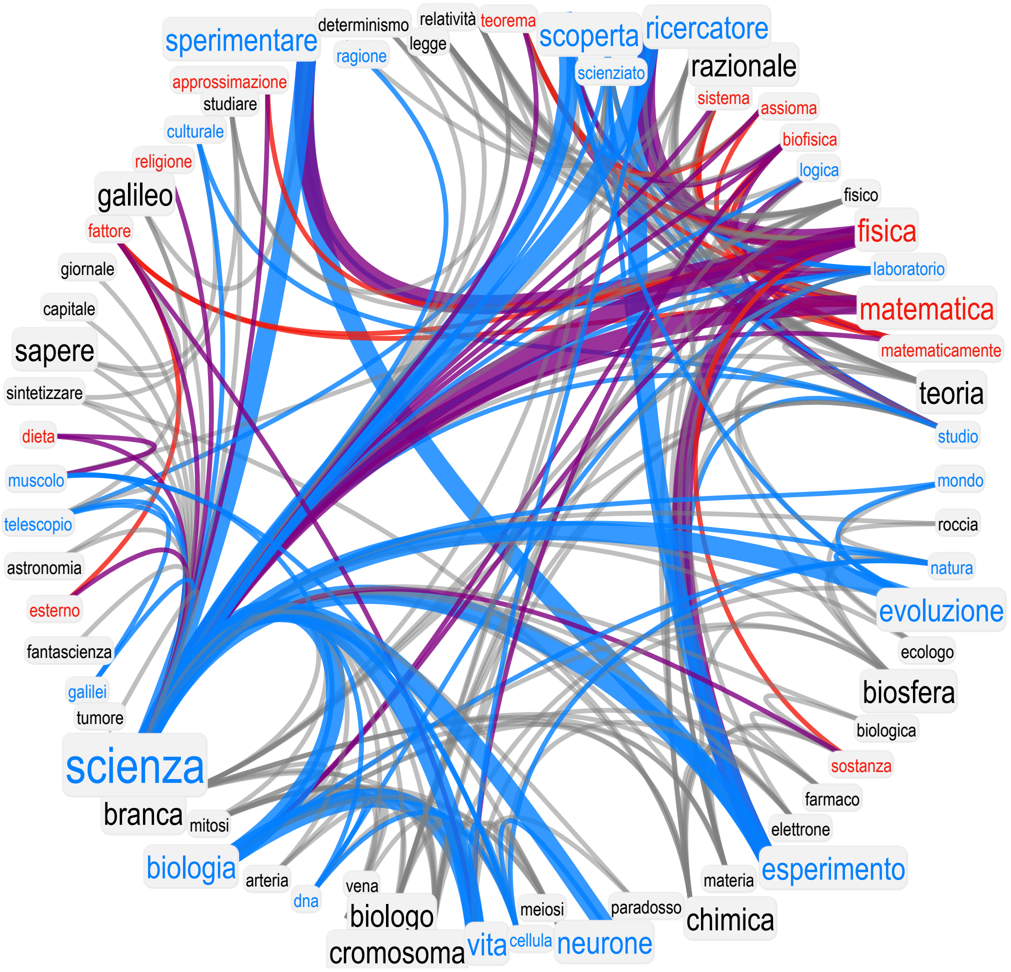

Fig 2. Including idiosyncratic associations highlights additional negative concepts semantically related to maths, physics and science. neighborhood of "science" as perceived by students including also idiosyncratic associations. Even in this case, most of the negative concepts associated to science are semantically related to maths and physics. The fact that the associations science-maths and science-physics indicates that students have the necessary knowledge for relating scientific discovery to quantitative STEM subjects. However the valence analysis clearly indicates a gap in diffusing the aura of positivity of science also to maths and physics.

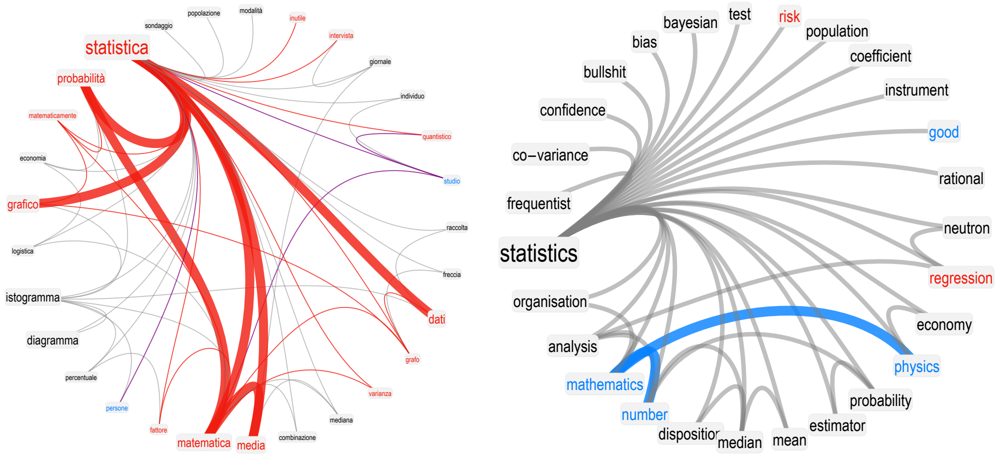

Fig 3. Also statistics is surrounded by a negative aura in students' FMN. Neighborhood of "statistics" as perceived by students (left) and researchers (right) including also idiosyncratic associations (thin links) and non-idiosyncratic associations (thicker links). Statistics is surrounded by a negative aura in the students' filtered FMN network, whereas researchers perceive it mainly as a neutral concept.
